# Transplantation of Bone Marrow Mesenchymal Stem Cells Prevents Radiation-Induced Artery Injury by Suppressing Oxidative Stress and Inflammation

**DOI:** 10.1155/2018/5942916

**Published:** 2018-02-28

**Authors:** Yanjun Shen, Xin Jiang, Lingbin Meng, Chengcheng Xia, Lihong Zhang, Ying Xin

**Affiliations:** ^1^Key Laboratory of Pathobiology, Ministry of Education, Jilin University, Changchun, China; ^2^Department of Pathology, Shanxi Medical University, Taiyuan, China; ^3^Department of Radiation Oncology, The First Hospital of Jilin University, Changchun, China; ^4^Department of Internal Medicine, Florida Hospital, Orlando, USA

## Abstract

The present study aims to explore the protective effect of human bone marrow mesenchymal stem cells (hBMSCs) on radiation-induced aortic injury (RIAI). hBMSCs were isolated and cultured from human bone marrow. Male C57/BL mice were irradiated with a dose of 18-Gy 6MV X-ray and randomly treated with either vehicle or hBMSCs through tail vein injection with a dose of 10^3^ or 10^4^ cells/g of body weight (low or high dose of hBMSCs) within 24 h. Aortic inflammation, oxidative stress, and vascular remodeling were assessed by immunohistochemical staining at 3, 7, 14, 28, and 84 days after irradiation. The results revealed irradiation caused aortic cell apoptosis and fibrotic remodeling indicated by aortic thickening, collagen accumulation, and increased expression of profibrotic cytokines (CTGF and TGF-*β*). Further investigation showed that irradiation resulted in elevated expression of inflammation-related molecules (TNF-*α* and ICAM-1) and oxidative stress indicators (4-HNE and 3-NT). Both of the low and high doses of hBMSCs alleviated the above irradiation-induced pathological changes and elevated the antioxidant enzyme expression of HO-1 and catalase in the aorta. The high dose even showed a better protective effect. In conclusion, hBMSCs provide significant protection against RIAI possibly through inhibition of aortic oxidative stress and inflammation. Therefore, hBMSCs can be used as a potential therapy to treat RIAI.

## 1. Introduction

Radiotherapy is an important treatment for malignant tumors. During the process, normal tissues surrounding the tumor would be irradiated and damaged. Therefore, when thoracic malignancies undergo radiotherapy, the thoracic aorta and other surrounding blood vessels are inevitably subject to radiation damage. Radiation-induced arterial injury (RIAI) was first reported in 1959 and considered as a chronic damage due to its insidious development for decades before the appearance of clinical symptoms [1]. Radiation exposure causes excessive production of eicosanoids, which are endogenous mediators of inflammatory reactions, such as vasodilation and vasoconstriction, increased vascular permeability and extravasation of leukocytes, microthrombus formation, and vascular endothelial apoptosis [2]. In large vessels, the main clinical manifestations of RIAI are atherosclerosis, stenosis, and obstruction [1]. It could occur in a variety of locations, including carotid artery [3], arteries of the upper limbs, axillary artery [4], and subclavian artery [5]. Previous studies have proved that the severity of large vessel injury was directly proportional to the dose and length of irradiation [6]. High-dose radiotherapy is a significant risk factor of accelerated carotid atherosclerotic disease [7].

Numerous clinical observations found that patients with RIAI suffered a lot and even died. For example, stroke cases were reported after radiotherapy to head and neck cancers [8]. Patients also suffered from angioplasty and stenting due to the radiotherapy-related artery stenosis and thrombosis [4]. However, the clinical drugs of glucocorticoid, antibiotics, and anticoagulant are only effective for symptomatic relief of RIAI but invalid for prevention. Therefore, it is urgent to find effective methods to prevent or alleviate RIAI-induced symptoms.

Mesenchymal stem cells (MSCs) are considered as important seed cells in regenerative medicine due to its powerful capacities of cytokines secretion, immune regulation, and multiple differentiation potential [9]. MSCs can be derived from many tissues, such as umbilical cord blood, placenta, muscle, adipose tissue, and bone marrow. Among these, MSCs from bone marrow have the highest proliferative capacity and keep their pluripotency even after 50 passages [10]. More and more studies indicated that MSCs had the beneficial effects on vascular injury [11, 12]. MSCs orchestrate the repair process of injured vessels by various mechanisms such as transdifferentiation, microvesicles or exosomes, and secreting cytokines [13, 14]. MSCs can directly differentiate into endothelial cells to participate in angiogenesis [15] or migrate and home to the injured large blood vessel for vascular repair by regulating various cell cytokines, such as transforming growth factor beta (TGF-*β*), vascular endothelial growth factor (VEGF), and intercellular cell adhesion molecules (ICAM) [16, 17].

Radiation exposure causes vascular endothelial dysfunction, which leads to vascular inflammatory and oxidative stress [18]. MSCs have been revealed to have the anti-inflammatory function in the repairing process of vascular injuries [19]. Recently, studies also proved that MSCs provide protection against radiation-induced liver injury and radiation-induced proctitis by antioxidative and anti-inflammatory process to maintain the vascular endothelial function [20, 21]. MSC treatment also protected lungs from radiation-induced endothelial cell loss and vascular damage by restoring antioxidant enzyme superoxide dismutase 1 expression [22]. Most importantly, clinical trials have reported that intravenous administration of allogeneic human bone marrow MSCs (hBMSCs) is safe for patients [23]. Based on these, cellular therapy of hBMSCs will be a potential approach to treat RIAI. However, there is no publication to observe the therapeutic effect of hBMSCs on RIAI.

Therefore, the present study is designed to apply intravenous administration of hBMSCs to an established RIAI mouse model so as to evaluate hBMSCs' potential protective role against RIAI. This study will provide evidence to use the human MSCs as a treatment for RIAI.

## 2. Materials and Methods

### 2.1. Isolation and culture of hBMSCs

The protocol used in this experiment was approved by the Ethics Committee of the College of Basic Medical Sciences of Jilin University (Changchun, China). Written informed consent was obtained from healthy volunteers with age from 18 to 45. Samples of human bone marrow were collected from healthy volunteers by lumbar puncture in The First Hospital of Jilin University (Changchun, China). The hBMSCs were isolated and cultured as described in previous studies [10]. Briefly, bone mononuclear cells were isolated from human bone marrow by density gradient centrifugation in a Percoll solution (1.073 g/ml, Pharmacia, USA). The isolated cells (P0) were cultured in Dulbecco's Modified Eagle's Medium containing 5.6 mmol/L glucose (DMEM) with 10% fetal bovine serum (FBS, Invitrogen, Carlsbad, CA). 48 h later, the medium was changed to wash off the nonadherent cells. 8–12 days later, individual colonies were selected, trypsinized, and replated as the first passage culture (P1). Cells were passaged every 3-4 days, and hBMSCs at the 5th passage (P5) were harvested for identification and transplantation *in vivo*.

### 2.2. Flow Cytometry Analysis

P5 hBMSCs were incubated for 1 h at 4°C with the following mouse anti-human antibodies (diluted at 1 : 100): CD105, CD73 (BD Biosciences, Bedford, MA), CD166, CD44, CD34, CD45, and CD31(Neo Marker, Fremont, CA) then incubated with secondary antibodies of CY3 or FITC (Abcam, Cambridge, MA) for 30 min at 4°C. hBMSCs were then analyzed using a FACS Calibur flow cytometer (BD Biosciences, San Jose, CA, USA).

For cell cycle analysis, 1 × 10^7^ hBMSCs at P5 were harvested, fixed in 70% ethanol for 20 min at 4°C, washed twice with PBS, and stained with 50 *μ*g/ml propidium iodide (PI, BD Biosciences) at 4°C for 30 min in the dark. Samples were analyzed by FACS Calibur using Cell Quest software in 24 h.

### 2.3. Immunofluorescent Staining

The P5 hBMSCs were fixed with 4% formaldehyde, treated with 3% H_2_O_2_, blocked in 1%BSA, then incubated with monoclonal antibodies against CD44, CD73, CD166, and CD105 (BD Biosciences, Franklin Lakes, NJ, USA, 1 : 1000 dilution) at 4°C overnight, and then incubated with IgG conjugated with fluorescence CY3 or FITC (1 : 200). Fluorescence signals were observed by laser scanning confocal microscopy (Olympus FV500, Japan).

### 2.4. Adipogenic, Osteogenic, and Chondrogenic Differentiation of hBMSCs

To evaluate the multilineage differentiation potential, the cells were induced to differentiation in adipogenic, osteogenic, or chrondrogenic medium for 2–4 weeks according to the manufacturer's protocol [24]. Lipid droplets in the cells were stained with Oil Red O solution. Calcium deposition was assessed by von Kossa, and chondrogenic differentiation was identified by Alcian blue staining.

### 2.5. Establishment of RIAI Mouse Models and Cell Transplantation

One hundred and forty male C57BL/6 mice, at 8 weeks of age, were purchased from Beijing Experimental Animal Technical Co. LTD. (Beijing, China). Mice were housed in the Animal Center of Jilin University (Changchun, China). All animal procedures were approved by the Animal Care and Use Committee of the Chinese Academy of Medical Sciences (Beijing, China). To establish RIAI model, mice were fixed in supine position after anesthesia with sodium pentobarbital and irradiated by 6MV X-ray of 18Gy once when mice lungs were shielded with lead sheaths. For hBMSC treatment, mice were given a tail vein injection of hBMSCs with a low dose of 10^3^ cells/g or a high dose of 10^4^ cells/g of body weight within 24 h after radiation. Mice serving as vehicle controls were given the same volume of PBS. Therefore, the mice were evenly divided into four groups (*n* = 7): the control group (control), the radiation group (IR), the radiation with low dose of the hBMSC group (IR + LD hBMSCs), and the radiation with high dose of the hBMSC group (IR + HD hBMSCs). At 3, 7, 14, 28, and 84 days after irradiation, mice were sacrificed with the heart perfusion of 4% phosphate-buffered formalin under anesthesia. Then the aortas were separated and fixed in 10% formalin. Each aorta of mice was average cut into 3 segments and embedded in one paraffin block and sectioned at 5 *μ*m thickness for histological studies.

### 2.6. Histopathological Examination

Hematoxylin and eosin (HE) staining was performed to examine the morphological changes and the thickness of the aortic wall. The thickness of aorta presented as width from intima to adventitia was measured by the Digimizer software in 30 randomly selected fields from 3 segments per aorta with total 7 mice in each group.

For immunohistochemical staining, the aortic paraffin sections were dewaxed, rehydrated, and incubated with citric acid buffer at 98°C for antigen retrieval, then with 3% hydrogen peroxide and 5% animal serum treatments. Those sections were incubated with primary antibodies against TGF-*β*, connective tissue growth factor (CTGF), ICAM-1, and tumor necrosis factor-*α* (TNF-*α*) at 1 : 300 dilution, heme oxygenase 1 (HO-1) and catalase at 1 : 200 dilution, 4- hydroxynonenal (4-HNE) at 1 : 400 dilution (all from Santa Cruz Biotechnology, Santa Cruz, CA), and 3-Nitrotyosine (3-NT) at 1 : 400 dilution (Millipore, Billerica, CA), overnight at 4°C. After being washed, sections were incubated with horseradish peroxidase-conjugated secondary antibodies (1 : 300–400 dilutions with PBS) and then treated with peroxidase substrate DAB kit (Vector Laboratories, Inc., Burlingame, CA) for the development of color and counterstained with hematoxylin.

The quantitative analyses of these immunohistochemical staining were achieved from 7 mice of each group. Three sections at an interval of 10 sections from each aorta (per mouse) were selected and at least five high-power fields were randomly captured in each section. Image Pro Plus 6.0 software was used to transfer the staining density in the area of interest to an integrated optical density (IOD), and the ratio of IOD/area in the experimental group was presented as a fold relative to that of control.

### 2.7. Apoptosis Assay

Apoptosis in the aorta was assessed by TUNEL assay using Peroxidase In Situ Apoptosis Detection Kit S7100 (Millipore, Billerica, MA), according to the manufacturer's instructions. Under the microscope, the cells with dark-brown nuclei were positive and counted in 30 random microscopic fields from 3 segments per aorta with total 7 mice per group. The results were presented as TUNEL positive cells relative to 100 cells.

### 2.8. Sirius Red Staining for Collagen

Sirius red staining for collagen accumulation was performed to examine aortic fibrosis. Sections were stained with 0.1% Sirius red F3BA and Mayer's Hematoxylin and then assessed for the presence of collagen using a Nikon Eclipse E600 microscopy system.

### 2.9. Statistical Analysis

Data are presented as the means ± standard deviation (SD, *n* = 7). Statistical evaluation was analyzed with SPSS 17.0 software. One-way ANOVA was performed to compare differences between groups, followed by pairwise repetitive comparisons using Tukey's test. Statistical significance was considered as *P* < 0.05.

## 3. Results

### 3.1. Morphology and Features of hBMSCs

**Figure 1:**
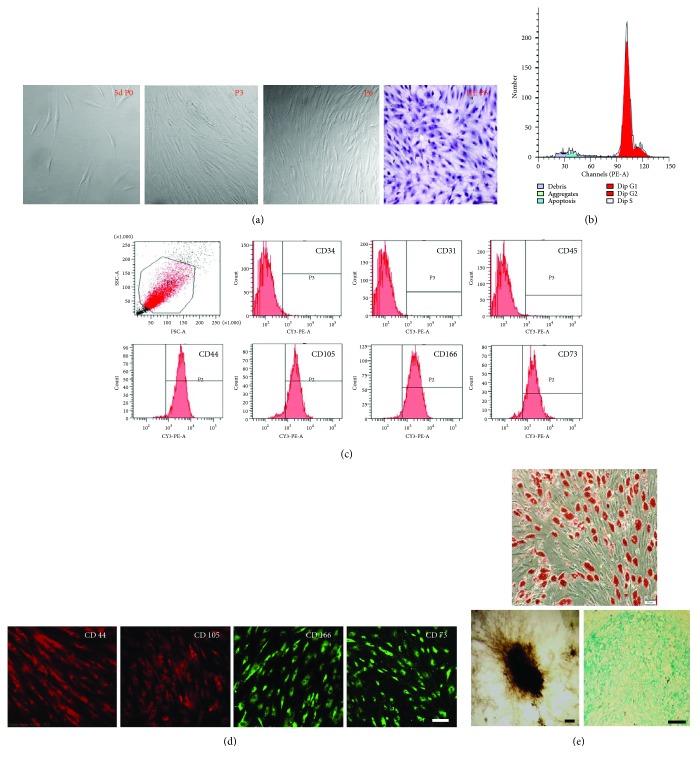
Morphology and features of human bone marrow mesenchymal stem cells (hBMSCs). (a) The morphological features of cultured hBMSCs at the 5th day; the 3rd and 6th passages (P3 and P6) were evaluated by the light microscope or HE staining. (b) Cell cycle analysis by FACS showed that (86.65% ± 2.8%) of P5 hBMSCs was in the G_0_/G_1_ phase and (14.35% ± 2.8%) was in the S + G_2_/M phase. (c) Flow cytometry analysis disclosed that more than 90% of P5 hBMSCs were positive for CD44, CD105, CD166, and CD73; however, they were negative for CD34, CD31, and CD45. (d) Immunofluorescence staining revealed that P5 hBMSCs expressed the antigens of CD73, CD44, CD105, and CD166. (e) hBMSCs differentiated into adipose cells that formed lipid droplets in the cytoplasm, indicated by positive Oil Red staining (upper). The differentiation of hBMSCs to bone was demonstrated by positive von Kossa staining (bottom left). The differentiation to cartilage was reflected by positive Alcian blue staining (bottom right). Scale bar, 50 *μ*m.

2 weeks after isolation and culture by density gradient centrifugation combined with individual colonies screening, P1 hBMSCs reached 80% confluence and then were passaged every 4-5 days for 9–12th passages without morphologic alteration. hBMSCs displayed fibroblast-like shape and homogenous and vortex-like growth in monolayers (Figure 1(a)). Cell cycle analysis revealed that P5 hBMSCs in quiescent phase of G_0_/G_1_ was 86.65 ± 2.8%, and in active proliferative phase of S + G_2_/M was 14.35 ± 2.8% (Figure 1(b)), with typical stem cell proliferation characteristics. Flow cytometry analysis of surface antigens on P5 hBMSCs showed that more than 90% of cells expressed CD44, CD73, CD166, and CD105, but less than 2% expressed CD34, CD31, and CD45 (Figure 1(c)). Immunofluorescence staining also confirmed these results (Figure 1(d)). Cultured in adipogenic medium for 2 weeks, P5 hBMSCs differentiated into adipogenic cells as shown by positive Oil Red O staining (Figure 1(e), upper). hBMSCs cultured in osteogenic medium for 3 weeks formed mineral deposits as demonstrated by positive von Kossa staining (Figure 1(e), down left). After induction for 3 weeks in chondrogenic medium, Alcian blue staining showed that hBMSCs expressed proteoglycan, an indicative of chondrogenic differentiation (Figure 1(e), down right). These results indicated that the cultured cells with relative homogeneity exhibited the characteristics of hBMSCs.

### 3.2. hBMSCs Alleviated Radiation-Induced Aortic Remodeling

**Figure 2:**
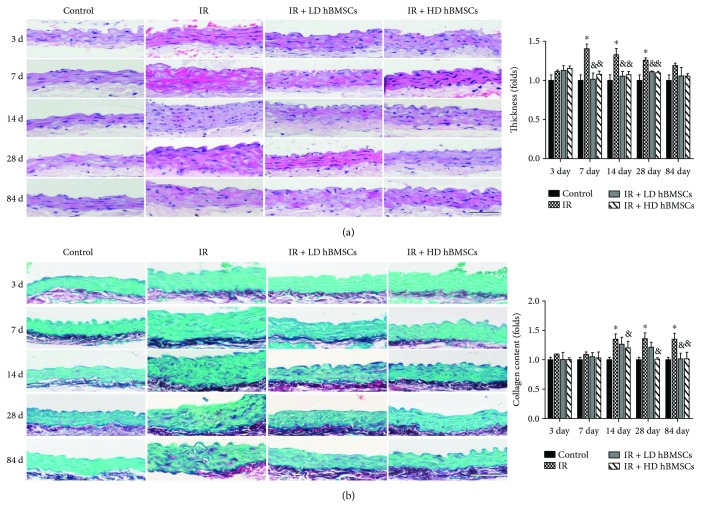
hBMSCs alleviated radiation-induced aortic pathological changes. Male C57BL/6 mice were irradiated by 6MV X-ray of 18Gy once with their lungs were shielded to establish the RIAI model. hBMSCs were injected by tail vein in a dose of 10^3^ or 10^4^ cells/g of body weight within 24 h after radiation. Therefore, the mice were evenly divided into four groups: the control group (control), the radiation group (IR), the radiation with low or high dose of the hBMSC group (IR + LD hBMSCs and IR + HD hBMSCs). At 3, 7, 14, 28, and 84 days after radiation, the aortas were isolated for histological studies. The pathological changes of aortas were examined by HE staining (a) and the accumulation of collagen was detected by Sirius red staining (b), followed by semiquantitative analysis. Data were presented as means ± SD (*n* = 7). ^∗^*P* < 0.05 versus control group; ^&^*P* < 0.05 versus IR group; ^#^*P* < 0.05 versus IR + LD hBMSC group. Scale bar, 50 *μ*m.

**Figure 3:**
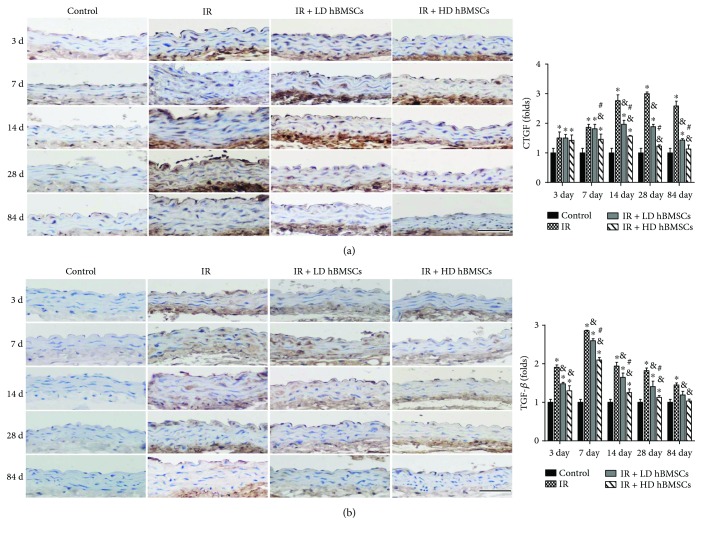
hBMSCs alleviated radiation-induced aortic fibrosis. Aortic fibrosis was examined by immunohistochemical staining for the expression of CTGF (a) and TGF-*β* (b), followed by semiquantitative analysis. Data were presented as means ± SD (*n* = 7). ^∗^*P* < 0.05 versus control group; ^&^*P* < 0.05 versus IR group; ^#^*P* < 0.05 versus IR + LD hBMSC group. Scale bar, 50 *μ*m.

Aortic pathological changes were firstly examined by H&E staining (Figure 2(a)), which displayed significantly increased tunica media thickness in the IR group mice at 7, 14, and 28 days after irradiation and slight-increased thickness at 84 days without significant difference, as compared with the controls. Meanwhile, low or high dose of hBMSC treatment could largely prevent those increased aortic thickening induced by radiation (Figure 2(a)) at each time point. Sirius red staining also revealed an increased collagen accumulation in aortic tunica media at 14, 28, and 84 days after exposure to 6MV X-ray (Figure 2(b)). High dose of hBMSC treatment significantly inhibited radiation-induced collagen accumulation in aortas on day 14, 28, and 84, while the inhibitory effect in low dose of the hBMSC treatment group was only observed on day 84. To further detect the effect of hBMSCs on radiation-induced aortic fibrosis, immunohistochemical staining for protein levels of profibrotic mediators, CTGF (Figure 3(a)) and TGF-*β* (Figure 3(b)) were measured. Compared with control mice, aortic CTGF and TGF-*β* levels in the IR group mice were all significantly increased on day 3, 7, 14, 28, and 84. Low dose of hBMSCs could prevent the increased CTGF expression in the aortas induced by radiation on day 14, 28, and 84, while the inhibitory effect of high dose of hBMSCs was observed as early as day 7 (Figure 3(a)). Increased aortic TGF-*β* expression induced by radiation was obviously suppressed by both low and high dose of hBMSC treatment at each time point (Figure 3(b)). Moreover, high dose of hBMSCs showed the stronger inhibitory effect on those two profibrotic mediators than low dose of hBMSCs (Figures 3(a) and 3(b)).

### 3.3. hBMSCs Reduced Radiation-Induced Aortic Inflammation

**Figure 4:**
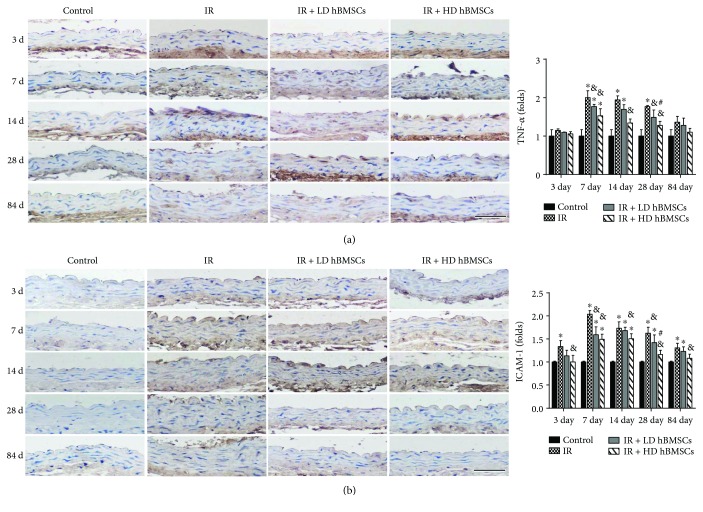
hBMSCs reduced radiation-induced aortic inflammation. Aortic inflammation was examined by immunohistochemical staining for the expression of TNF-*α* (a) and ICAM-1 (b), followed by semiquantitative analysis. Data were presented as means ± SD (*n* = 7). ^∗^*P* < 0.05 versus control group; ^&^*P* < 0.05 versus IR group; ^#^*P* < 0.05 versus IR + LD hBMSC group. Scale bar, 50 *μ*m.

Previous studies have suggested sustained inflammatory response occurs in irradiated human arteries [25]. Regarding inflammation as the primary risk factor for vascular endothelium remodeling, the protein levels of TNF-*α* (Figure 4(a)) and ICAM-1 (Figure 4(b)) were examined by immunohistochemical staining. Compared to the control group, aortic TNF-*α* expression in the IR group mice was significantly increased on day 7 and then progressively decreased. The difference between the two groups was still remarkable until day 28. Low or high dose of hBMSC treatment prevented increased TNF-*α* expression in IR groups (Figure 4(a)). It was also noticed that ICAM-1 expression in aortas was significantly increased at 3, 7, 14, 28, and 84 days after exposure to X-ray. This increase was significantly reduced by high dose of hBMSC treatment. The inhibitory effect of low dose of hBMSCs on ICAM-1 expression was only observed on day 7 and 28 (Figure 4(b)).

### 3.4. hBMSCs Attenuated Radiation-Induced Aortic Oxidative Damage

**Figure 5:**
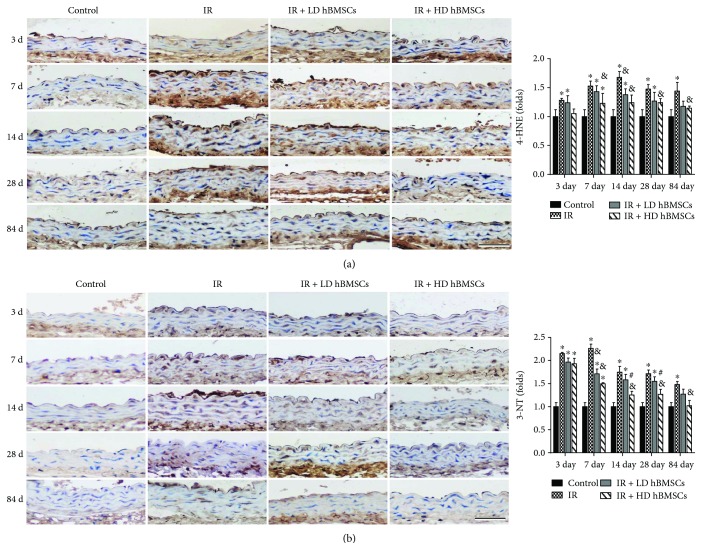
hBMSCs attenuated radiation-induced aortic oxidative damage. Aortic oxidative damage was examined by immunohistochemical staining for the expressions of 4-HNE (a) and 3-NT (b), followed by semiquantitative analysis. Data were presented as means ± SD (*n* = 7). ^∗^*P* < 0.05 versus control group; ^&^*P* < 0.05 versus IR group; ^#^*P* < 0.05 versus IR + LD hBMSC group. Scale bar, 50 *μ*m.

Oxidative damage was detected by examining the accumulation of 4-HNE (Figure 5(a)) and 3-NT (Figure 5(b)) as indices of lipid peroxidation and protein nitration, respectively. Results of immunohistochemical staining showed a significant accumulation of 3-NT and 4-HNE in the aortas of irradiated mice on day 3, 7, 14, 28, and 84. High dose of hBMSC treatment significantly inhibited the radiation-induced expression of 3-NT and 4-HNE from 7 days to 84 days, while low dose of hBMSCs showed the inhibitory effect on 4-HNE only at day 14, as well as on 3-NT at day 7. On day 14 and 28, high dose of hBMSCs shows a stronger inhibitory effect on 3-NT (Figure 5(b)) than low dose of hBMSCs, while no difference was observed on 4-HNE expression between those two groups.

### 3.5. hBMSCs Reduced Radiation-Induced Aortic Cell Apoptosis

**Figure 6:**
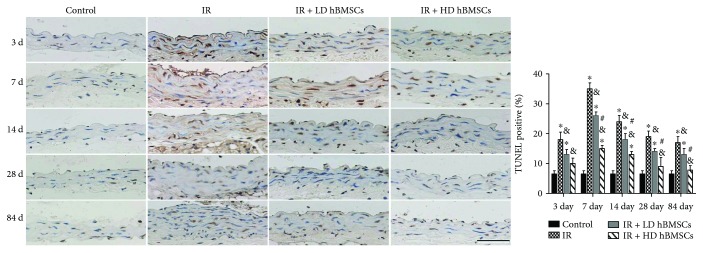
hBMSCs reduced radiation-induced aortic apoptosis. The apoptotic cell was examined by TUNEL staining followed with semiquantitative analysis. Data were presented as means ± SD (*n* = 7). ^∗^*P* < 0.05 versus control group; ^&^*P* < 0.05 versus IR group; ^#^*P* < 0.05 versus IR + LD hBMSC group. Scale bar, 50 *μ*m.

Effect of radiation and hBMSCs on aortic cell apoptosis was evaluated by TUNEL staining (Figure 6). The results showed that cell apoptosis in aortas of irradiated mice was significantly increased compared with that in control mice on day 3, 7, 14, 28, and 84 but was reduced by low or high dose of hBMSC treatment. Moreover, compared with the LD hBMSC group, HD hBMSCs revealed a stronger inhibitory effect on radiation-induced aortic cell apoptosis, indicated by a lower TUNEL positive ratio on day 7, 14, 28, and 84 (Figure 6).

### 3.6. hBMSC Upregulated Antioxidant Enzymes Expression of HO-1 and Catalase in Aortas

**Figure 7:**
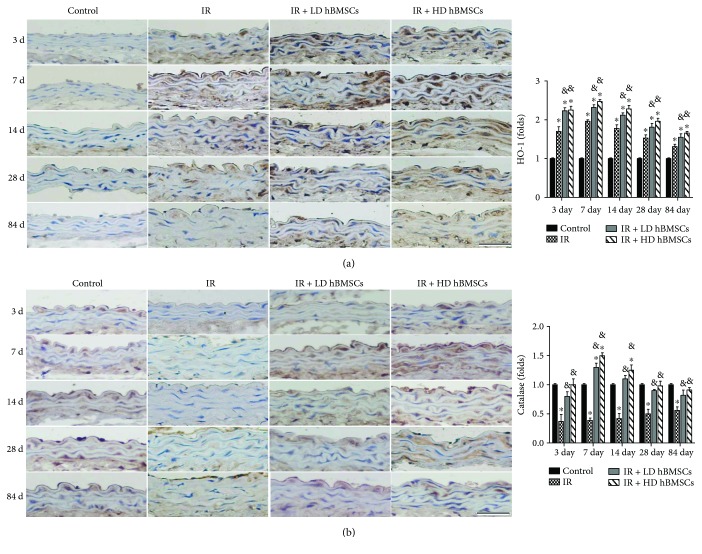
hBMSC upregulated antioxidant enzymes expression of HO-1 and catalase in aortas. The antioxidant enzyme expression of HO-1 (a) and catalase (b) was examined by immunohistochemical staining followed with semiquantitative analysis. Data were presented as means ± SD (*n* = 7). ^∗^*P* < 0.05 versus control group; ^&^*P* < 0.05 versus IR group; ^#^*P* < 0.05 versus IR + LD hBMSC group. Scale bar, 50 *μ*m.

Since hBMSCs attenuated radiation-induced aortic oxidative damage (Figure 5), whether this protective effect of hBMSCs on the aorta is associated with upregulation of antioxidant enzymes was examined first by measuring HO-1 and catalase expression with immunohistochemical staining (Figure 7). The results showed that compared with the control group, HO-1 expression was significantly increased in the aorta of the IR group and hBMSC group mice at each time point (Figure 7(a)). There was a further increase of the HO-1 expression in the aorta of low and high dose of hBMSC treatment mice compared with the IR group (Figure 7(a)). Moreover, it was shown that catalase expression in the aorta of irradiated mice was significantly decreased compared with that in control mice on day 3, 7, 14, 28, and 84 but was significantly elevated by low or high dose of hBMSC treatment (Figure 7(b)).

## 4. Discussion

In the present study, we have explored for the first time the protective effects of bone marrow mesenchymal stem cells on radiation-induced pathological changes and damage in the aorta. We demonstrated the establishment of RIAI mouse model by evaluating the aortic thickening, fibrotic remodeling, inflammation, oxidative stress and cell apoptosis. We showed low or high dose of hBMSC treatment can partially reverse radiation-induced pathologic changes in aortas and the high dose of hBMSCs has even a better protective effect.

Based on the ability to adhere to plastic culture dishes, MSCs derived from human adult bone marrow of healthy donors were selected [24]. In our study, to get more uniform hBMSCs, individual colonies were selected and expanded after the original seeding for 8–12 days. The flow cytometry analysis showed that the cultured P5 cells expressed mesenchymal stem cell markers of CD73, CD105, CD44, and CD166 but were negative for hematopoietic stem/progenitor cell marker of CD34, endothelial cell marker of CD31, and leukocyte cell marker of CD45. The cell cycle analysis demonstrated that more than 86% of P5 cells were in quiescent phase (G_0_/G_1_phase). Meanwhile, the isolated cells exhibited their capacity to undergo adipogenic, chondrogenic, and osteogenic differentiation (Figure 1). These results fully confirmed the obtained hBMSCs were highly homogenous and pluripotent and therefore could be used as seed cells for the following experiments.

Previous studies have investigated irradiation to carotid arteries of ApoE^−^/^−^mice induced inflammatory and thrombotic responses *in vivo* with various radiation doses [26]. Based on those references, C57/BL mice were radiated with a single dose of 18Gy X-ray to establish RIAI mice models in our study. RIAI in our mice model was successfully developed, indicated by significant increases of aortic remodeling and cell apoptosis, as well as aortic, inflammation, and oxidative stress.

MSCs have been reported to repair the injured vascular wall [12] and play a local immunomodulation on injured rat carotids [11]. Yang et al. also confirmed that BMSC transplantation through tail vein injection promotes angiogenesis and VEGF expression in rats [27]. However, previous studies also revealed that different doses of MSCs could exert different effects *in vivo*. Appropriate dose of MSCs was required for successful transplantation and improvement of functional properties [28]. In addition, a higher incidence of adverse events may occur in a high-dose MSC treatment. For example, intravenous administration of a high-dose MSC (5.0 × 10^5^ and 1.0–3.0 × 10^6^ cells/mice) induced a lethal portal vein or pulmonary embolism [29, 30]. Therefore, the doses of 1 × 10^3^ and 1 × 10^4^ cells/g of mice body weight (approximate to 2.0 × 10^4^ and 2.0 × 10^5^ cells/g) were chosen in the present study, which were also supported by a previous study [31]. Similarly, no embolism and related death were observed in the present study. Our data demonstrated that both doses of hBMSCs had partially prevented the radiation-induced aortic injury, including the aortic cell apoptosis, fibrotic remodeling, inflammation, and oxidative stress. Moreover, the higher dose of hBMSCs showed more remarkable protective effects, implied by the less aortic cell apoptosis (Figure 6) and lower expression of CTGF and TGF-*β* (Figure 3).

Vascular inflammation is one of the prominent features of radiation-induced tissue injury [32]. The effects of inflammation include induction of oxidative stress, cell apoptosis, and endothelial dysfunction, all of which could contribute to the structural and functional abnormalities of the blood vessel [33]. Recent studies have revealed that endothelial cells were injured shortly after radiotherapy [34]. It is widely believed that radiation upregulates proinflammatory cytokines and adhesion molecules in endothelial cells of injured blood vessels [25, 35]. Consistent with those findings, we observed that the expression of ICAM-1, an adhesion molecule, as well as TNF-*α*, a proinflammatory cytokine, was significantly increased in aortas as early as 3 days and 7 days after irradiation, respectively, and kept at the high levels until 84 days or 28 days (Figure 4). These results indicated that both ICAM-1 and TNF-*α* were involved in the radiation-induced aortic inflammatory injuries. It is reported that inflammation was observed in early stage of irradiated arteries [26]. TNF-*α* was shown to enhance ICAM-1 on activated endothelial cells in the artery inflammation disease [36]. Therefore, at 84 days of later postirradiation, the expression of TNF-*α* in irradiated aortas was no difference with the control, while the expression of ICAM-1was still kept higher than the control (Figure 4). It has also been reported that rat MSCs play an immunomodulatory role via diminishing secretion of inflammation-related molecules CXCL1 and ICAM-1 to accelerate reparation of abnormal arteries [37]. BMSC inhibits TNF-*α* production of anti-inflammatory and antifibrosis in lung injury [38]. In addition, the study by Forte et al. also disclosed that MSCs inhibited inflammatory response to facilitate endothelial reparation [11]. Consistent with this, our present study found that hBMSCs diminished radiation-induced increase of TNF-*α* and ICAM-1 expressions in aortas, which illustrated that hBMSCs had an effect on diminishing radiation-induced aortic inflammation.

Inflammation and oxidative stress are reciprocal causes and outcomes, both of which are main pathogenic factors for the development of various cardiovascular diseases [39]. It has been well established that irradiation causes radiolysis of intracellular water molecules, leading to increased production of ROS. In addition, inflammatory cytokines can induce reactive oxygen species (ROS)/reactive nitrogen species (RNS) production in the vascular system [40]. Extra generation of those species or insufficient endogenous antioxidant defenses results in oxidative stress in the organs. The vascular endothelium is a major target of oxidant stress [41]. Endothelial dysfunction is described as the initial pathogenic event of radiation-injured vascular injury. Vascular oxidative stress contributes to vascular dysfunction [42]. This study showed that accompanied with increased expressions of inflammation-related molecules TNF-*α* and ICAM-1, the markers of oxidative stress (4-HNE and 3-NT) were significantly upregulated in aortas of RIAI mice. Meanwhile, hBMSC administration, especially the high dose of hBMSCs, significantly inhibited the accumulation of 3-NT and 4-HNE in aortas from 7 days to 84 days after radiation (Figure 5). These results implied the antioxidant effect of hBMSCs, which was in accord with a previous report that BMSC administration attenuated hepatic ischemia-reperfusion injury by suppressing oxidative stress and apoptosis in rats [43].

To further detect the potential mechanism of antioxidant effect of hBMSCs, the antioxidant enzyme expression of HO-1 and catalase was observed in the aorta. It was found that radiation significantly induced the HO-1 expression (Figure 7(a)) and attenuated the catalase expression (Figure 7(b)) in the aortas, in agreement with those observations on radiation-induced lung and hematopoietic system injury [44–46]. Based on vascular disease studies, HO-1 has shown the beneficial effects on the endothelium [47] and plays an antioxidant effect on vascular injury [48]. Catalase, as an H_2_O_2_ scavenging enzyme, has been found to be protective against vascular endothelial oxidative damage [49]. Prior studies showed that MSCs could resort the radiation-induced low activity of antioxidant enzymes, including catalase [50]. Consistent with those observations, our study found that the low or high dose of hBMSC treatment further enhanced the upregulation of HO-1 and reversed the decrease of catalase induced by the radiation in the aorta (Figure 7). These findings indicated that hBMSCs possibly suppresses ROS generation by upregulating expression of related antioxidant enzymes. However, there may be a concern that hBMSCs further increased the expression of HO-1 compared with the IR group (Figure 7(a)). We speculate that irradiation as a stress stimulating antioxidant reaction including the increase of HO-1 expression is an adaptive response. This adaptive response tries to provide certain protections but is not sufficient to completely prevent the progression of aortic pathological changes. However, upregulated levels of HO-1 in hBMSC-treated RIAI mice are high enough to efficiently reduce radiation-induced oxidative damage, as we observed here. This speculation was also supported by the observations of astaxanthin's protection on irradiation-induced hematopoietic system injury [44] and antioxidant MG132 on diabetes-induced aortic oxidative damage [51].

Radiation-induced vascular fibrosis is a complex and dynamic process, which is initiated and aggravated by proinflammatory and profibrotic cytokines and oxidative stress. Arteries injured by radiation could easily develop spontaneous atherosclerosis [52], which was associated with the increased inflammation and fibrinogen [53]. Studies have proved that high dose of radiation can induce vascular fibrosis [54] and TGF-*β*, a profibrotic cytokine, which plays a critical role in the process of radiation-induced vascular smooth muscle cells fibrosis [55]. Connective tissue growth factor (CTGF) is induced by TGF-*β* and also contributes to collagen synthesis and fibroblast proliferation [56]. Therefore, suppression of TGF-*β* and CTGF may be sufficient to prevent radiation-induced aorta remodeling. Expectedly, our study found the expressions of TGF-*β* and CTGF in aortas were all significantly increased from day 3 to 84 after radiation (Figure 3), accompanied with increased collagen accumulation in aortic tunica media (Figure 2) from day 14 to 84. hBMSC administration partially prevented the aortic fibrosis and remodeling, reflected by the complete suppression of increased TGF-*β* expression and partial inhibition of CTGF expression, as well as aortic collagen accumulation (Figures 2 and 3).

The acute phase of vascular injury occurs within hours to weeks after irradiation is characterized by endothelial swelling, apoptosis, and vascular permeability and edema [57]. This phase is often accompanied by an inflammatory reaction, leading to tissue edema [58]. As time goes on, secretion of inflammatory factors and inflammatory response was gradually decreased. Later vascular injury appears weeks to months postirradiation and includes thickening of basement membranes, collagen deposition, fibrosis, and scar [59]. According to this, we observed that inflammation-related cytokines of ICAM-1 and TNF-*α*, as well as aortic cell apoptosis, were increased in aortas as early as 3 days and reached their peaks at 7 days after irradiation. While the radiation-induced vascular fibrosis reflected by the collagen accumulation appeared at 14 days, later than aortic inflammation and cell apoptosis. Meanwhile, the tunica media thickness in the IR group mice after irradiation was significantly increased at 7, 14, 28, days and without a significant difference at 84 days, as compared with the controls. Therefore, it is suspected that the increased thickness of aortas at 7 days of postirradiation was mainly caused by inflammatory exudation and tissue edema, and at 14 and 28 days, it was by inflammation combined with collagen accumulation. At a later stage of 84 days, slight increased aortic thickness without significant difference in the IR group was probably due to less inflammation. However, aortic thickness in hBMSC treatment groups was always kept at the normal level.

More and more evidences show that BMSCs could directly differentiate into vascular endothelial cells and smooth muscle cells, even forming functional vessels [15, 60]. However, reports suggest that differentiation either by transdifferentiation or cell fusion appears too low to explain the significant improvement of vascular repair [61]. Based on this, we had not focused on the transdifferentiation of hBMSCs to vascular cells in this study but observed the expression of vascular damage-related cytokines. Recent studies have shown the key mechanism by which MSCs enhance tissue function is through its paracrine functions. For example, Ortiz et al. report that BMSC inhibits TNF-*α* production by the secretion of the IL-1 receptor agonist [38]. MSCs induced an increase in antioxidant gene expression of Nrf2, which reduce ROS production decreasing oxidative stress induced by irradiation in the injured liver [20]. Extracellular vesicles derived from MSCs protect against acute kidney injury through antioxidation by enhancing Nrf2 activation [62] and against experimental colitis by suppressing the apoptosis via reducing the apoptotic genes of caspase-3, 8, and 9 in rats [63]. Considering the facts that hBMSCs significantly diminished the expressions of proinflammatory molecules (TNF-*α* and ICAM-1) and profibrotic cytokines (TGF-*β* and CTGF) in the present study, as well as the report of its paracrine activity on attenuating inflammation, oxidative stress, and apoptosis [63], we speculate that hBMSCs facilitated aortic repair mainly through paracrine actions, without largely depending on direct differentiation.

There may be also a couple of limitations of the present study. Although publications have demonstrated that MSCs could migrate and home to the injured large blood vessel for vascular repair [16, 17], the location, transdifferentiation, and protective mechanism of MSCs in the irradiated aortas have not been directly observed. Parameters, strictly correlated with the endothelial function, such as vasorelaxation and nitric oxide production, as well as vascular permeability, have not been assessed in the present study. And it is uncertain whether the aortic injury caused by a prolonged radiotherapy can be prevented by hBMSCs. Thus, further experiments are needed to clarify the unknown.

In conclusion, hBMSC administration alleviated radiation-induced aortic injuries indicated by attenuated aortic thickening, fibrotic remodeling, and cell apoptosis. We considered the protective effect of hBMSCs is mainly through the suppression of radiation-induced oxidative stress and inflammation, including downregulation of TNF-*α*, ICAM-1, TGF-*β*, and CTGF as well as upregulation of antioxidant enzymes HO-1 and catalase. Therefore, hBMSCs may be a promising therapeutic approach to treat RIAI.
